# Dickkopf‐1 drives perineural invasion via PI3K–AKT signaling pathway in head and neck squamous cancer

**DOI:** 10.1002/mco2.518

**Published:** 2024-03-22

**Authors:** Jingyi Wang, Qianying Li, Faya Liang, Xin Du, Pan Song, Taowei Wu, Renhui Chen, Xiaorong Lin, Qinglian Liu, Hai Hu, Ping Han, Xiaoming Huang

**Affiliations:** ^1^ Department of Otolaryngology‐Head and Neck Surgery Sun Yat‐sen Memorial Hospital Sun Yat‐sen University Guangzhou China; ^2^ Guangdong Provincial Key Laboratory of Malignant Tumor Epigenetics and Gene Regulation Guangzhou China; ^3^ Department of Oncology, Sun Yat‐sen Memorial Hospital Sun Yat‐sen University Guangzhou China; ^4^ Diagnosis and Treatment Center of Breast Diseases Shantou Central Hospital Shantou China

**Keywords:** AKT, Dickkopf 1, head and neck squamous cell carcinoma, perineural invasion

## Abstract

Perineural invasion (PNI) leads to the poor prognosis of head and neck squamous cancer (HNSCC) patients, but the mechanism of PNI remains unclear. Dickkopf‐1 (DKK1), a secretory protein in the Wnt signaling pathway, was found indeed upregulated in HNSCC cells and tissues. Higher expression of DKK1 was statistically relevant to T stage, N stage, PNI, and poor prognosis of HNSCC. DKK1 overexpression enhanced the migration abilities of cancer cells. Moreover, DKK1‐overexpressing cancer cells promoted cancer cells invasion of peripheral nerves in vitro and in vivo. Mechanistically, DKK1 could promote the PI3K–AKT signaling pathway. The migration abilities of neuroblastoma cells, which were enhanced by DKK1‐overexpressing HNSCC cell lines, could be reversed by an inhibitor of Akt (MK2206). The association of DKK1 with PNI was also confirmed in HNSCC samples. Variables, including T stage, N stage, DKK1 expression, and PNI, were used to establish a nomogram to predict the survival probability and disease‐free probability at 3 and 5 years. In summary, DKK1 can promote the PI3K–AKT signaling pathway in tumor cells and then could induce neuritogenesis and facilitate PNI. MK2206 may be a potential therapeutic target drug for HNSCC patients with PNI.

## Graphical abstract

Perineural invasion (PNI) leads to poor prognosis in head and neck cancer patients. Dickkopf 1 (DKK1) is associated with PNI and was statistically relevant to T stage, N stage, PNI, and poor prognosis of HNSCC. DKK1 can promote the PI3K–AKT signaling pathway in tumor cells and then could induce neuritogenesis and facilitate PNI. MK2206 may be a potential therapeutic target drug for HNSCC patients with PNI.

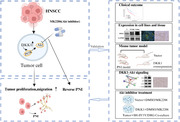

## 1 BACKGROUND

Head and neck squamous cell carcinoma (HNSCC) is the seventh most common malignant tumor worldwide, and the incident of it still continue to rise with 1.1 million new cases worldwide.^1^ It is a complex disease with a profound impact on patients’ life qualify.^2^ HNSCC originates from mucosal epithelial cells and mainly occurs in the upper aerodigestive tract, such as the oral cavity, oropharynx, nasopharynx, larynx, and hypopharynx.^1^, ^3^, ^4^ With the development of chemotherapy and the widespread use of immunotherapy, the prognosis of HNSCC is still poor, with approximately 500,000 deaths worldwide in 2016.^1^ The 5‐year overall survival (OS) rate of patients with locoregionally advanced HNSCC is only 40%.^1^ Thus, it is of great significance to investigate the potential pathogenesis and new therapeutic strategies for HNSCC.

In previous study, perineural invasion (PNI), which is a process of cancer cells invading the perineural sheath, was observed in many HNSCC cases.^5^ Because of the special anatomical location of HNSCC, HNSCC can invade into motor nerves, sensory nerves, and even brain, which may lead to pain and/or some cranial neuropathies, such as voice hoarseness and loss of cutaneous and mucosal sensation.^5^ The prognosis of HNSCC patients with PNI is very poor, and the occurrence of PNI always leads to shortened 5‐year disease‐free survival (DFS), tumor progression, marginal invasion, and so forth.^6^, ^7^ Furthermore, the American Joint Committee on Cancer (AJCC) guidelines classified HNSCC patients with PNI as stage T3, indicating that PNI is of great significance for the treatment and prognosis of HSNCC patients.^8^ However, the mechanism of PNI is still unclear, and there are no specific treatment strategies for patients with PNI. Therefore, identifying the key genes and molecular mechanisms of PNI are expected to provide a theoretical basis for the discovery of molecular markers of PNI in HNSCC.

Dickkopf‐1 (DKK1) is a secretory protein in the Wnt signaling pathway, and its high expression may lead to the aberrant activation of noncanonical Wnt signaling (β‐catenin independent pathway) by activating the pathway downstream of cytoskeleton‐associated protein 4 (CKAP4) and inhibiting LDL receptor‐related protein 6 (LRP6).^9^ DKK1 has been reported to be highly expressed in various malignant tumors, such as breast cancer, esophageal cancer, and pancreatic cancer, and is associated with poor prognosis.^9^, ^10^, ^11^, ^12^ However, the specific function of DKK1 in HNSCC with PNI remains unknown.

In our study, we found that PNI was common in HNSCC patients and was associated with poor prognosis. DKK1 was highly expressed in HSNCC patients and could promote tumor progression in vitro and in vivo. It also induced neuritogenesis and facilitated PNI via PI3K–AKT signaling pathways. DKK1 may be a potential treatment target for HNSCC with PNI.

## 2 RESULTS

### 2.1 PNI leads to poor prognosis in HNSCC patients

**FIGURE 1:**
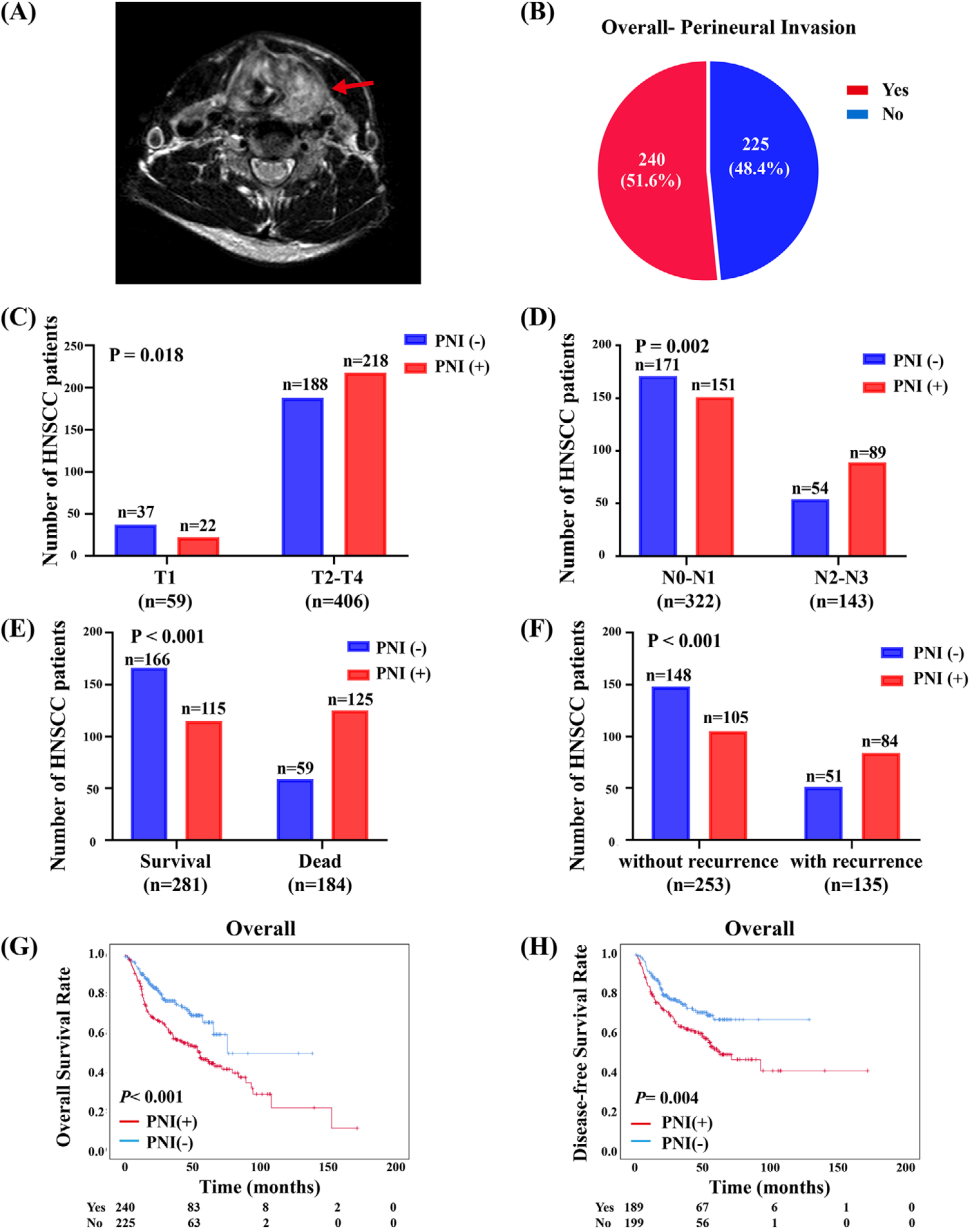
PNI correlates with poor prognosis of HNSCC. (A) Magnetic resonance imaging (MRI) demonstrating direct tumor extension to the nerve (red arrow: nerve). (B) The proportion of PNI among HNSCC patients in the TCGA database and our cohort. (C–F) The associations of PNI and patients’ clinicopathologic features, including T stage, N stage, survival state, and DFS. (G and H) Kaplan–Meier survival analysis showing the OS (G) and DFS (H) of HNSCC patients with or without PNI among HNSCC patients in the TCGA database and our cohort (log‐rank test).

To explore the relationship of HNSCC and nerve, we referred to magnetic resonance imaging (MRI). MRI showed that HNSCC was closer to the nerve, which might lead to perineural invasion more easily (Figure 1A). To determine the incidence of PNI in HNSCC patients, we analyzed the TCGA database and our cohort and found that approximately half of the HNSCC patients had PNI (Figure 1B and Figure S1A,B). Moreover, there was a strong association with PNI and patients’ clinicopathologic features, including T stage (*p* = 0.018), N stage (*p* = 0.002), OS (*p* < 0.001), and DFS (*p* < 0.001) (Figure 1C–F).

To further explore how PNI affected the prognosis of HNSCC patients, we performed Kaplan–Meier survival analysis. As expected, in our cohort, the cumulative 3‐year overall survival (OS) and disease‐free survival (DFS) rates were 93.0% and 86.0% in the PNI‐negative group and 83.5% and 71.8% in the PNI‐positive group, respectively (*p* < 0.05; Figure S1C,D). Taken together, the TCGA database and our cohort showed that PNI‐positive patients had a lower OS rate and DFS rate (*p* < 0.05; Figure S1E,F and Figure 1G,H).

**FIGURE 2:**
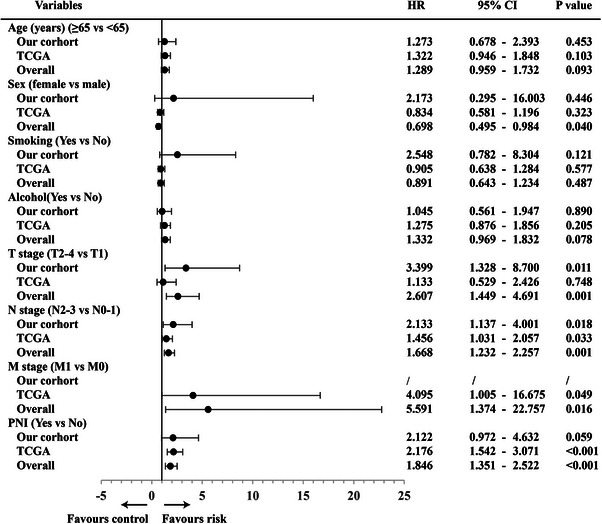
Forest plots of the univariate Cox regression analysis of different variables corresponding to OS in different cohorts. The univariate Cox regression analysis showing that T stage, N stage, M stage, and PNI were the prognostic factors of HNSCC.

Next, we performed univariate analyses with the Cox proportional hazards model to determine whether the PNI was a prognostic predictor. Many variables, including age, sex, smoking, alcohol, T stage, N stage, M stage, and PNI, were assessed in the model to test their relationship with OS. The univariate Cox regression analysis showed that T stage, N stage, M stage, and PNI could be regarded as prognostic factors of HNSCC in the overall cohort (Figure 2). Variables that were most significantly associated with OS or DFS in the univariate analysis were assessed with a forward stepwise model in the multivariable Cox regression analysis. The multivariable analysis results showed that the PNI was an independent prognostic factor for OS (*p* = 0.001) and DFS (*p* = 0.015) in HNSCC patients (Table 1). These results demonstrated that PNI played an important role in HNSCC and was associated with poor prognosis.

**TABLE 1:** Multivariable analysis of OS and DFS of HNSCC patients in TCGA database and our cohort.

Variables (*n* = 465)	OS			DFS		
	HR	95% CI	*p*‐Value	HR	95% CI	*p*‐Value
**T stage: T2+T3+T4/T1**	2.310	1.276–4.184	0.006^*^	/	/	/
**N stage: N2+N3/N0+N1**	1.458	1.073–1.980	0.016^*^	1.638	1.146–2.340	0.007^*^
**M stage: M1/M0**	9.549	2.302–39.607	0.002^*^	/	/	/
**PNI: Yes/No**	1.720	1.252–2.364	0.001^*^	1.552	1.089–2.211	0.015^*^

* *p* < 0.05.

### 2.2 DKK1 is overexpressed in most HNSCC cells and tissues

**FIGURE 3:**
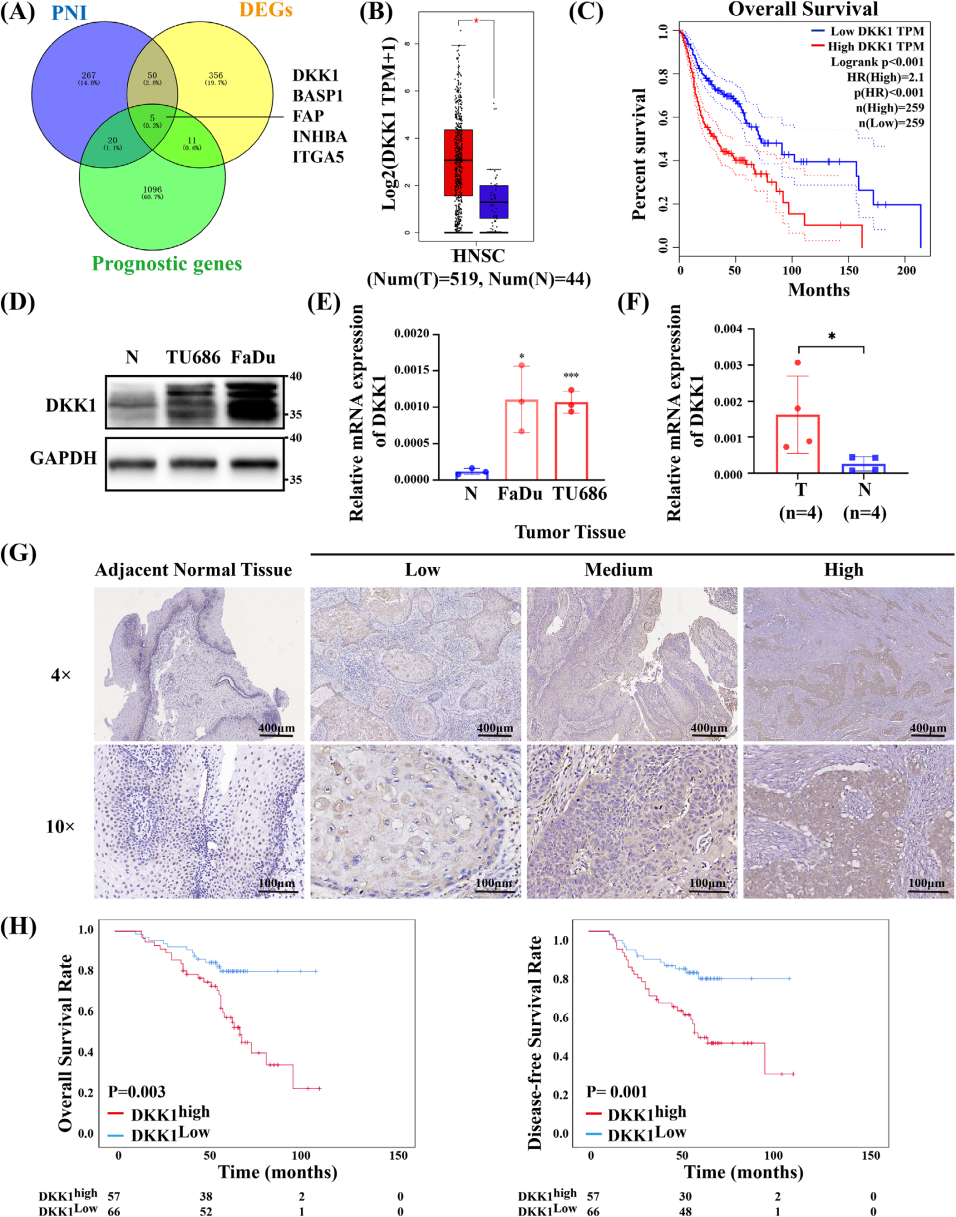
DKK1 is elevated in HNSC and correlates with a poor prognosis of HNSCC. (A) Venn diagram showing the IDEGs from TCGA databases. Blue area: genes associated with PNI; yellow area: genes upregulated in tumors; green area: associated with HNSCC patients’ prognosis. (B) Average expression level of DKK1 in TCGA databases analyzed by GEPIA tools. (C) Kaplan–Meier survival analyzed by GEPIA tools showing the overall survival (OS) of HNSCC patients with low or high DKK1 expression among HNSCC patients in TCGA database (log‐rank test). (D) Western blot showing the expression of DKK1 in the HNSCC cell lines (FaDu and TU686) and normal squamous epithelial cells. (E) Real‐time PCR showing the expression of DKK1 in the HNSCC cell lines (FaDu and TU686) and normal squamous epithelial cells. (F) Real‐time PCR showing the expression of DKK1 in the HNSCC samples (*n* = 4) and controls (*n* = 4). (G) Representative IHC results of different levels of DKK1 protein in tumor tissues and adjacent normal tissues. (H) Kaplan–Meier survival analysis showing the OS and disease‐free survival (DFS) of HNSCC patients with low or high DKK1 expression among HNSCC patients in our cohort (log‐rank test). Data information: All error bars, mean values ± SD, and *p*‐values were determined by unpaired two‐tailed Student's *t*‐test of *n* = 3 independent biological experiments. *p < 0.05, **p < 0.01, ***p < 0.001; ns, no significance.

To further determine the molecular mechanism of PNI in HNSCC, we compared HNSCC samples with PNI and without PNI in the TCGA database and found that 342 genes were differentially expressed according to the Venn diagram (Figure 3A). Subsequently, the intersecting DEGs (IDEGs) were screened for detailed analysis using the Venn diagram, and five genes, including DKK1, BASP1, FAP, INHBA, and ITGA5, were screened (Figure 3A). BASP1, FAP, INHBA, and ITGA5 had been reported to be upregulated in several cancers and could regulate neuronal activity,^13^, ^14^, ^15^, ^16^, ^17^ but their relationship with DKK1, nerves, and HNSCC still remains unknown.

We further cultivated the expression of DKK1 in HNSCC and found that DKK1 was not only upregulated significantly in HNSCC comparing with the matched TCGA normal and GTEx data (Figure 3B), but was also most strongly associated with shorter OS time (Figure 3C). To examine the expression of DKK1 in HNSCC cell lines, we performed real‐time PCR and Western blot in normal squamous epithelial cells (Figure S2A) and HNSCC cell lines. HNSCC cell lines, including TU686 and FaDu, had higher DKK1 expression at both the mRNA and protein levels (Figure 3D,E). We further performed real‐time PCR in four cancer tissues and four noncancerous epithelial tissues, and found that DKK1 was highly expressed in HNSCC (*p* = 0.0473; Figure 3F). Thus, DKK1 may be an important gene in HNSCC.

### 2.3 DKK1 is correlated with poor prognosis in HNSCC patients

Then, IHC was performed to detect the protein expression levels of DKK1 in HNSCC samples. As a secreted protein, DKK1 was localized mainly in cytoplasm and few of them were localized in nucleus (Figure 3G). We further analyzed the association between DKK1 expression and the clinical characteristics of HNSCC patients. A total of 46.3% (57/123) of HNSCC patients had high DKK1 expression (Table 2). DKK1 expression was significantly related to T stage (*p* < 0.001), N stage (*p* < 0.001), clinical stage (*p* < 0.001), survival state (*p* < 0.001), and perineural invasion (*p* < 0.001) in HNSCC patients (Table 2). According to the univariate and multivariable Cox regression analysis results, DKK1 expression was considered an independent prognostic factor for OS (*p* = 0.029) and DFS (*p* = 0.027) in HNSCC patients (Table 3).

**TABLE 2:** Correlation between the expression of DKK1 and the clinicopathologic features of HNSCC.

	All cases (*n* = 123)	DKK1 expression		*p*‐Value
		Low (*n* = 66)	High (*n* = 57)	
Age (years)				0.367
<65	79	40 (60.6%)	39 (68.4%)	
≥65	44	26 (39.4%)	18 (31.6%)	
Smoking				0.493
No	18	11 (16.7%)	7 (12.3%)	
Yes	105	55 (83.3%)	50 (87.7%)	
Alcohol				0.147
No	54	25 (37.9%)	29 (50.9%)	
Yes	69	41 (62.1%)	28 (49.1%)	
Gender				1.000
Female	5	3 (4.50%)	2 (3.50%)	
Male	118	63 (95.5%)	55 (96.5%)	
T stage				<0.001^*^
T1	38	29 (43.9%)	9 (15.8%)	
T2	33	23 (34.8%)	10 (17.5%)	
T3	28	7 (10.6%)	21 (36.8%)	
T4	24	7 (10.6%)	17 (29.8%)	
N stage				<0.001^*^
N0	73	51 (77.3%)	22 (38.6%)	
N1	18	6 (9.10%)	12 (21.1%)	
N3	32	9(13.6%)	23 (40.4%)	
M stage				/
M0	123	66 (100%)	57 (100%)	
M1	0	0 (0.0%)	0 (0.0%)	
TNM stage (UICC/AJCC 8th edition, 2018)				<0.001^*^
I	33	29 (43.9%)	4 (7.0%)	
II	16	13 (19.7%)	3 (5.3%)	
III	27	12 (18.2%)	15 (26.3%)	
IV	47	12 (18.2%)	35 (61.4%)	
State				<0.001^*^
Survival	82	54 (81.8%)	28 (49.1%)	
Death	41	12 (18.2%)	29 (50.9%)	
Recurrent				<0.001^*^
No	79	52 (78.8%)	27 (47.4%)	
Yes	44	14 (21.2%)	30 (52.6%)	
Perineural invasion				<0.001^*^
No	43	37 (56.1%)	6 (10.5%)	
Yes	80	29 (43.9%)	51 (89.5%)	

^a^ Fisher's exact test.

* *p *< 0.05.

**TABLE 3:** Univariate analysis and multivariable analysis of OS and DFS in HNSCC in our cohort.

Variables	OS			DFS		
	HR	95% CI	*p*‐Value	HR	95% CI	*p*‐Value
**Univariable analysis (*n* = 123)**							
**Age: <65/≥65**	1.273	0.678–2.393	0.453	0.889	0.476–1.660	0.711
**Smoking: Yes/No**	2.548	0.782–8.304	0.121	2.667	0.825–8.620	0.101
**Alcohol: Yes/No**	1.045	0.561–1.947	0.890	1.242	0.677–2.278	0.485
**Gender: Women/Man**	2.173	0.295–16.003	0.446	1.929	0.265–14.038	0.516
**T stage: T2+T3+T4/T1**	3.399	1.328–8.700	0.011*	2.757	1.226–6.199	0.014*
**N stage:N2+N3/N0+N1**	2.133	1.137–4.001	0.018*	2.930	1.596–5.379	0.001*
**PNI: Yes/No**	2.122	0.972–4.632	0.059	2.285	1.096–4.767	0.028*
**DKK1 expression: High/Low**	2.699	1.371–5.311	0.004*	2.716	1.437–5.134	0.002*
**Multivariable analysis (*n* = 123)**						
**T stage: T2+T3+T4/T1**	2.631	1.001–6.914	0.050*	/	/	/
**N stage: N2+N3/N0+N1**	/	/	/	2.237	1.176–4.255	0.014*
**DKK1 expression: High/Low**	2.169	1.083–4.344	0.029*	2.140	1.090–4.202	0.027*

* *p *< 0.05.

The cumulative 3‐year OS and DFS rates of our cohort were 86.9% and 76.9%, respectively. The cumulative 3‐year OS and DFS rates were 80.4% and 66.1% in the high DKK1 expression group, whereas 92.4% and 86.3% in the low DKK1 expression group, respectively (*p* < 0.01; Figure 3H).

These results demonstrated that DKK1 could be considered a carcinogenetic gene related to tumor progression and poor prognosis in HNSCC patients.

### 2.4 DKK1 overexpression plays an important role in PNI in HNSCC patients

**FIGURE 4:**
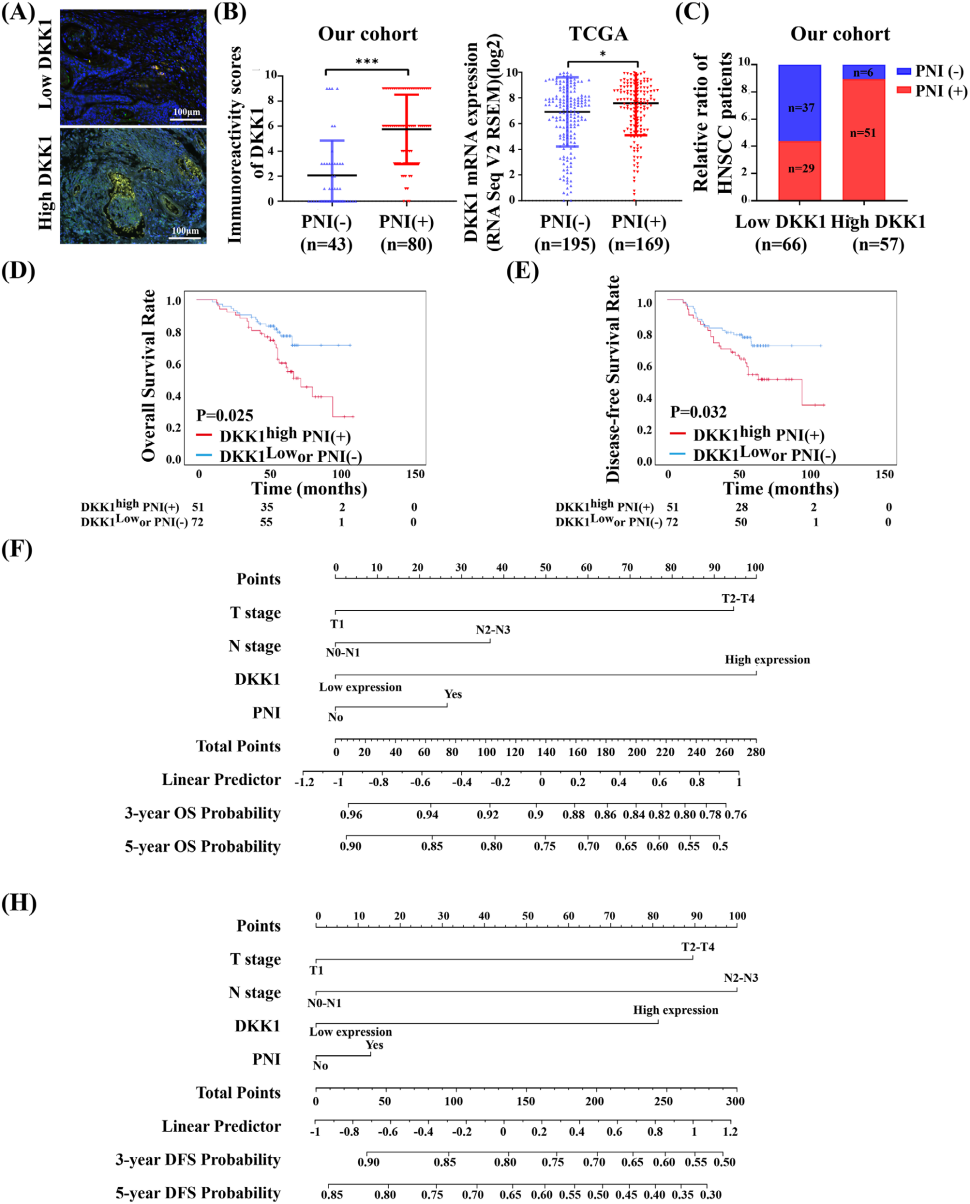
DKK1 overexpression plays an important role in PNI in HNSCC patients. (A) Representative IF images of S100 in low or high DKK1 expressing HNSCC tissues. (B) The expression of DKK1 in HNSCC samples with or without PNI in our cohort and TCGA databases. (C) The relative ratio of HNSCC patients of PNI in low or high DKK1 expression group. (D and E) Kaplan–Meier survival analysis showing OS (D) and DFS (E) of HNSCC patients with or without DKK1^high^ PNI (+) among HNSCC patients in our cohort (log‐rank test). (F–H) Nomogram, including T stage, N stage, DKK1 expression, and PNI for 5‐year OS (F) and 5‐year DFS (H) predicting 3‐ and 5‐year survival probability and disease‐free probability in patients with HNSCC. *p < 0.05, **p < 0.01, ***p < 0.001

To determine whether DKK1 overexpression played a role in PNI in HNSCC patients, we further used an anti‐S100 antibody to perform immunofluorescence (IF), and found that DKK1 overexpression obviously induced PNI (Figure 4A). In our cohort, HNSCC samples with PNI had higher immunoreactivity scores (IRS) for DKK1, and in the TCGA database, DKK1 expression was higher in samples with PNI according to the RNA‐seq (Figure 4B). Similarly, DKK1 overexpression increased the likelihood of PNI (Figure 4C). Furthermore, we performed Kaplan–Meier survival analysis to determine the cumulative 3‐year OS and DFS rates of our cohort of 123 HNSCC patients. The cumulative 3‐year OS and DFS rates were 82.0% and 70.0% in the DKK1^high^ PNI^(+)^ group, whereas 90.3% and 81.9% in the DKK1^low^ or PNI^(‐)^ group, respectively (*p *< 0.05; Figure 4D,E).

PNI and the expression of DKK1 played an important role in the prognosis of HNSCC, and we tried to build a nomogram for prognostic prediction based on these factors. Variables, including T stage, N stage, DKK1 expression, and PNI, were used to establish a nomogram to predict the survival probability and disease‐free probability at 3 and 5 years (Figure 4F–H). This predictive model may help predict the prognosis of HNSCC.

### 2.5 DKK1 promotes tumor progress

**FIGURE 5:**
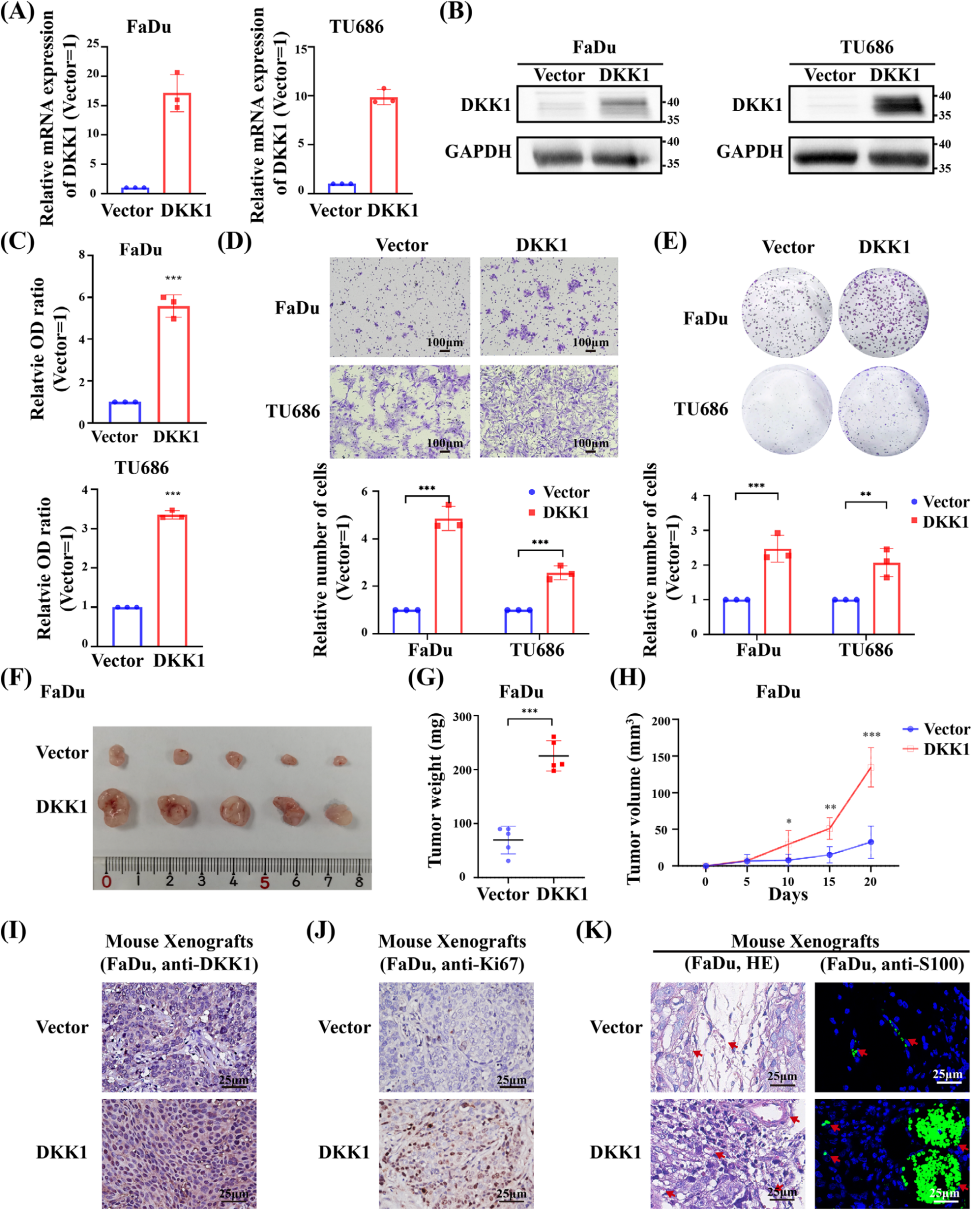
DKK1 promotes HNSCC cell migration in vitro and in vivo. (A–C) Establishment of stable cell lines (FaDu, TU686) overexpressing DKK1, as confirmed by real‐time PCR (A), Western blot (B), and ELISA (C). (D) Representative images of migration assays of vector‐ or DKK1‐overexpressing HNSCC cell lines, respectively. The results are from three different experiments. Number of migrated cells per field of view are plotted. (E) Representative images of colony formation of vector‐ or DKK1‐overexpressing HNSCC cell lines, respectively. The results are from three different experiments. Number of migrated cells per field of view are plotted. (F–H) Xenograft tumor growth of DKK1 overexpression FaDu stable cell lines in nude mice. Images of tumors (F), tumor weight (G), tumor volume (H) of two groups. *n* = 5 mice per group. (I and J) Representative IHC results of DKK1 (I) and Ki67 (J). (K) H&E staining and IF results showing the existence of PNI in DKK1 overexpressing group (red arrows: peripheral nerve). *p < 0.05, **p < 0.01, ***p < 0.001.

To investigate the function of DKK1 in the progression of HNSCC, HNSCC cell lines, including FaDu and TU686, were used to establish stable DKK1‐overexpressing cell lines. The cell lines were then confirmed by real‐time PCR (Figure 5A), Western blot (Figure 5B), and ELISA (Figure 5C). Transwell assays showed that overexpressing DKK1 could enhance the migration potency of the FaDu and TU686 cell lines (Figure 5D). Cell proliferation was also promoted by the overexpression of DKK1, as confirmed by colony formation assays (Figure 5E).

To further explore the effect of DKK1 on the tumorigenicity of HNSCC cells in vivo, DKK1‐overexpressing cells and control cells were subcutaneously injected into nude mice. We found that exogenous tumors injected with DKK1‐overexpressing cells were larger (Figure 5F) and heavier (Figure 5G) than those injected with control cells. The growth rate was enhanced in the DKK1‐overexpressing group (Figure 5H). Moreover, IHC analysis showed that DKK1 expression was increased (Figure 5I) and the Ki67‐positive rate (Figure 5J) was increased in the DKK1‐overexpressing subcutaneous xenografts. Furthermore, HE staining and IF also showed an interesting phenomenon in which the subcutaneous peripheral nerves of nude mice were invaded by tumors in the DKK1‐overexpressing groups (Figure 5K). All these results indicated that DKK1 could not only promote cancer cell proliferation and migration abilities but also may induce PNI.

### 2.6 DKK1 induces PNI in vitro and in vivo

**FIGURE 6:**
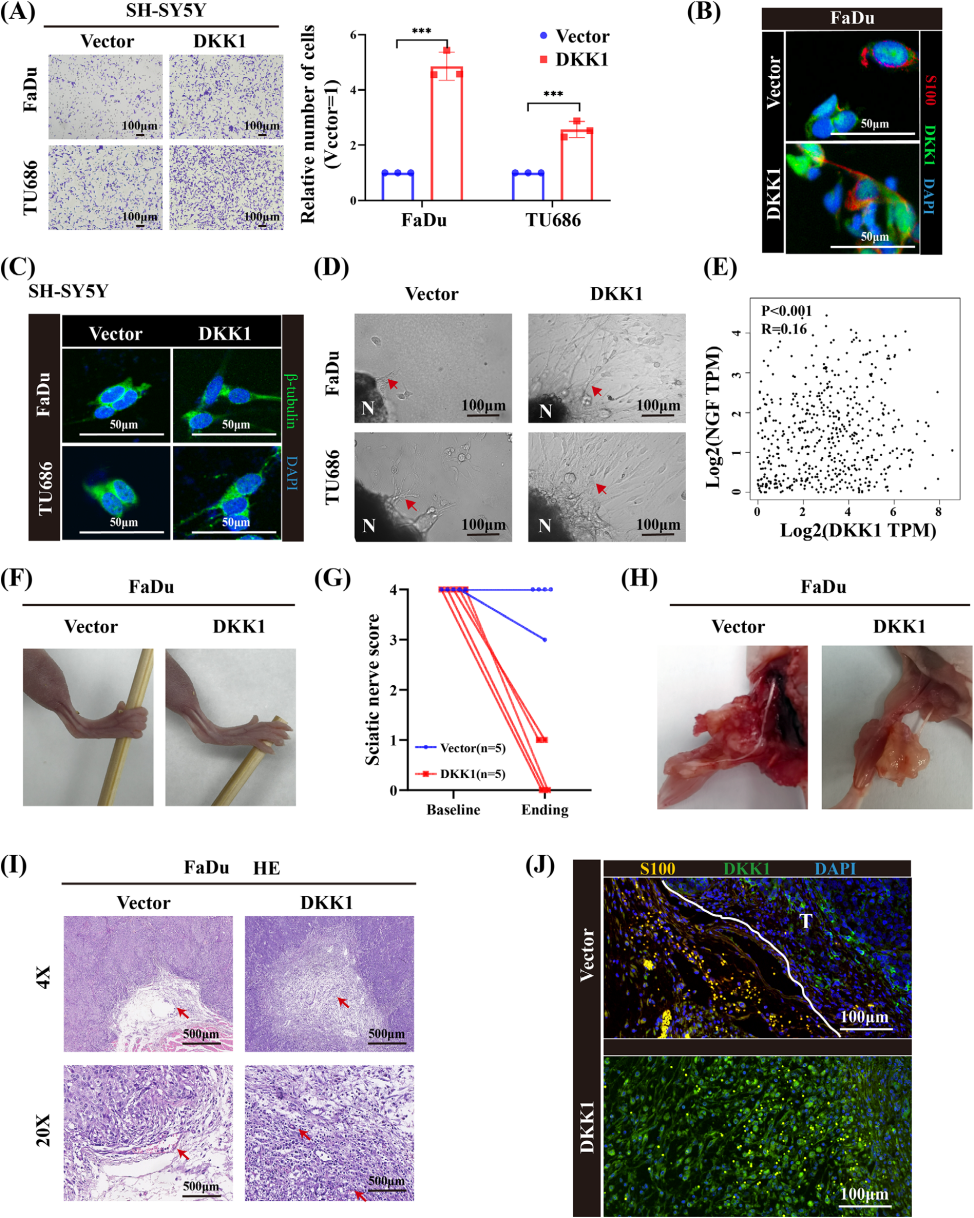
DKK1 promotes PNI in vitro and in vivo. (A) Representative images of migration assays of SH‐SY5Y neuroblastoma cells cocultured with vector‐ or DKK1‐overexpressing HNSCC cell lines. The results are from three different experiments. The number of migrated cells per field of view are plotted. (B and C) Representative images of IF showing neuritogenesis of SH‐SY5Y neuroblastoma cells induced by DKK1‐overexpressing HNSCC cell lines. (D) Representative images showing neurite outgrowth of DRG induced by DKK1‐overexpressing HNSCC cell lines (red arrows: neurite outgrowth of DRG). (E) The correlation between DKK1 and NGF analyzed by GEPIA tools. (F and G) Representative images of hindlimb paralysis (F) and sciatic nerve scores (G) of mice 2 weeks after tumor implantation in the DKK1‐overexpressing group compared with the control. (H) Representative images of PNI in mice. (I) H&E staining results confirming the existence of PNI in the DKK1‐overexpressing group (red arrows: peripheral nerve). (J) Representative images of IF staining DKK1 and S100 in the mouse xenograft tumors.***p < 0.001.

To investigate the function of DKK1 in promoting PNI, we cocultured HNSCC and SH‐SY5Y neuroblastoma cells. Transwell assays showed that overexpressing DKK1 in cancer cells could promote the migration of SH‐SY5Y cells (Figure 6A). After coculture of SH‐SY‐5Y cells with overexpressing DKK1 or control HNSCC cells, neuritogenesis and commingling of neuroblastoma cells were increased in the DKK1‐overexpressing group (Figure 6B,C). Moreover, to verify that DKK1 promotes neuritogenesis, the dorsal root ganglia (DRG) was separated and cocultured with HNSCC. As shown, overexpressing DKK1 cancer cells induced more neuritogenesis than control cells (Figure 6D). It had been reported that neural growth factor neurotrophins (NGF) were related to PNI.^18^, ^19^ We found a statistical correlation between DKK1 and NGF by using GEPIA2 tools (Figure 6E). These findings indicated that DKK1 could promote the interactions of nerve and tumor cells.

To further clarify the interactions of cancer cells and nerves in vivo, we established a model of nerve invasion in which cancer cells were implanted in a distal part of the sciatic nerve. After 2 weeks, all the mice in the DKK1‐overexpressing group developed hindlimb paralysis and could not grasp the swab (Figure 6F), and the sciatic nerve scores were lower in the DKK1‐overexpressing group (Figure 6G). All mice were sacrificed, and the sciatic nerves were dissected. As shown, tumors invaded the nerve in the DKK1‐overexpressing group, while tumors in the control group only grew along the nerve without invasion (Figure 6H). HE staining showed that nerves were invaded by cancer cells in the DKK1‐overexpressing group (Figure 6I). Co‐staining of DKK1 and S100 in the mouse xenograft tumors showed that the cancer cells invaded the nerve in DKK1‐overexpressing group (Figure 6J). These in vivo results indicated that DKK1 could promote HNSCC invasion of peripheral nerves.

### 2.7 DKK1 mediated PNI in HNSCC via PI3K–AKT signaling pathway

**FIGURE 7:**
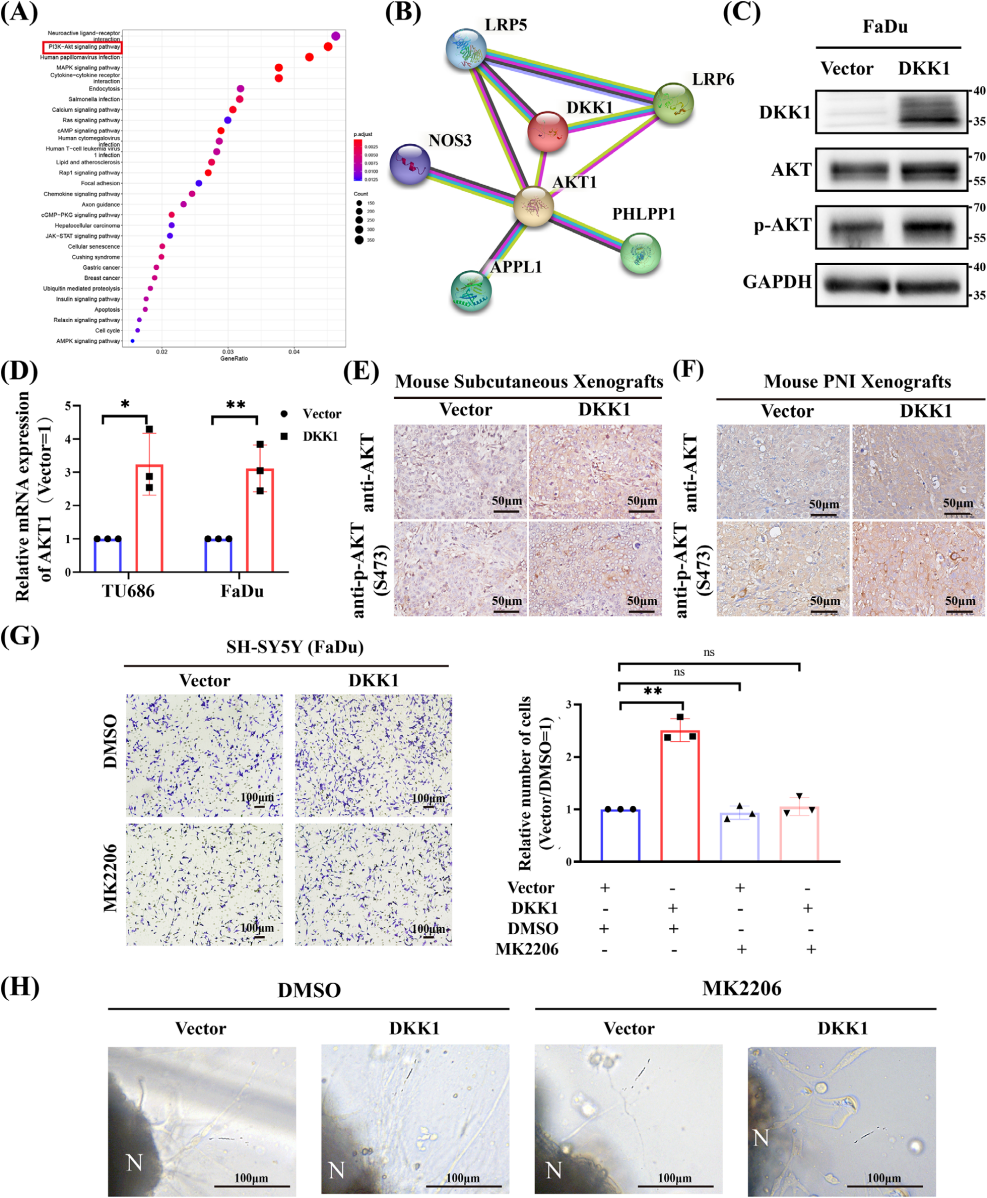
DKK1 regulates HNSCC cell migration and PNI through PI3K–AKT signaling pathways. (A) KEGG analysis of signaling pathways regulated by PNI in HNSCC. (B) Protein‒protein interaction (PPI) network related to DKK1 analyzed by the STRING database. (C) Western blot showing DKK1 promoting the expression of AKT and p‐AKT (FaDu). (D) Real‐time PCR showing Akt1 upregulation by DKK1 in HNSCC cell lines (FaDu, TU686). (E and F) Representative IHC results of AKT and p‐AKT (S473) in mouse xenografts. (G) Representative images of migration assays of SH‐SY5Y neuroblastoma cells cocultured with vector or DKK1‐overexpressing HNSCC cell lines after incubation with DMSO or MK2206 (10 nM). The results are from three different experiments. The number of migrated cells per field of view are plotted. (H) Representative images showing neurite outgrowth of DRG induced by DKK1‐overexpressing or vector HNSCC cell lines after incubation with DMSO or MK2206 (10 nM).*p < 0.05, **p < 0.01, ***p < 0.001.

To clarify the potential mechanism of DKK1‐mediated PNI, Kyoto Encyclopedia of Genes and Genomes (KEGG) showed that PNI primarily affected several signaling pathway gene sets, including neuroactive ligand‒receptor interaction and phosphoinositide 3‐kinase (PI3K)–Akt signaling pathway (Figure 7A). Then, we identified proteins related to DKK1 in the STRING database, and the protein‒protein interaction (PPI) network in Figure 7B shows the relationship between Akt1 and DKK1. The GEPIA2 tool showed the statistical correlation between DKK1 and Akt1 as well as Akt1 and GDNF/NGF (Figure S2B,C). Western Blot and real‐time PCR further showed that DKK1 could potentiate the mRNA and protein expression of Akt1 (Figure 7C,D and Figure S2D). Moreover, IHC analysis showed that AKT and p‐AKT were increased in both subcutaneous xenografts and PNI xenografts of nude mice stably overexpressing DKK1 (Figure 7E,F). Therefore, the PI3K–AKT signaling pathway might be a downstream pathway of DKK1 that plays an important role in the PNI process in HNSCC.

To cultivate whether AKT inhibitor could be used as a therapeutic target, we used MK2206, which was an inhibitor of Akt for further study. We found that MK2206 could reverse the migration abilities of DKK1‐overexpressing HNSCC cell lines (Figure S2E). MK2206 could reverse the migration abilities of SH‐SY5Y cells, which was enhanced by DKK1‐overexpressing HNSCC cell lines (Figure 7G). Furthermore, MK2206 impaired neuritogenesis inducing by DKK1‐overexpressing HNSCC cell lines (Figure 7H). Collectively, these results suggested that DKK1 may be a potential treatment target for HNSCC with PNI.

## 3 DISCUSSIONS

PNI refers to the infiltration of tumor cells along the nerve and/or nerve sheath, and is an interactive symbiotic process between nerves and cancer cells, which is a tumor metastasis mode.^19^ It mainly occurs in tumors near the nerves and is always associated with function loss, pain, numbness, and other discomfort symptoms.^20^ In the 19th century, some scholars proposed the concept of PNI by finding HNSCC growing along nerves and infiltrating into the intracranial space.^21^ Since then, PNI has been reported in a variety of malignant tumors, including pancreatic cancer, colorectal cancer, and uterine cancer, and is associated with local recurrence and poor prognosis.^22^, ^23^, ^24^ However, the potential mechanism of PNI is still unclear.

It has also been reported that HNSCC cancer cells can secrete neurotrophins and neurotransmitters such as NGF and GDNF.^21^ These neurotrophins can stimulate the communication of nerve and cancer cells and induce axonogenesis and neurogenesis in cancers.^19^, ^25^ Tumor cells can express galanin receptors and receive the stimulation of galanin produced by neuronal cells, which can activate various cancer‐promoting mechanisms.^5^ These results suggest that the occurrence of PNI promotes the development of malignant tumors. According to the TCGA database and our cohort, we found that approximately half of HNSCC patients had PNI. Patients with PNI were associated with poor prognosis, which might lead to treatment escalation. The mutational status in HNSCC may lead to poor prognosis.^26^ Unfortunately, because this was a retrospective study, we were unable to obtain the specific mutations of the patients. In our future study, we will cultivate relationship of the mutational status and PNI.

There are still some difficulties in CT/MRI images to determine PNI, and there is a lack of standardization of pathological diagnosis. Therefore, in our study, we found that DKK1 may be a biomarker predicting PNI through the analysis of the TCGA database. DKK1 is an antagonist of the canonical Wnt signaling pathway (β‐catenin‐dependent pathway), but recent reports found that high DKK1 expression may lead to the aberrant activation of Wnt signaling and could activate the noncanonical Wnt signaling pathway in cancers.^9^ However, the association between DKK1 and HNSCC clinical characteristics is unclear.

To detect the role of DKK1 in HNSCC, we performed IHC, and our results showed that high DKK1 expression was associated with T stage, N stage, OS, DFS, and nerve invasion. DKK1 was also an independent prognostic factor in predicting HNSCC OS and DFS. Our study further found that overexpressed DKK1 could enhance the proliferation and migration abilities of cancer cells in vitro and in vivo. All these results indicated that DKK1 was also associated with the malignant progression of HNSCC.

According to the above clinical analysis, we found that DKK1 was associated with nerve invasion. However, the role and mechanism of DKK1 in the occurrence of PNI in HNSCC have not been reported. To verify this hypothesis, we cocultured HNSCC and SH‐SY5Y neuroblastoma cells and found that DKK1 enhanced nerve cell migration. In addition, our study also found that cancer cells overexpressing DKK1 were more invasive toward DRG and induced more neuritogenesis. In vivo study also showed that overexpressing DKK1 can promote HNSCC invasion toward peripheral nerves. Moreover, in in vivo tumorigenesis, the subcutaneous peripheral nerves of nude mice were also invaded by tumors in the DKK1‐overexpressing groups. These findings showed the important function of DKK1 in PNI.

In breast cancer, DKK1 can be stimulated by the upstream RSPO2–RANKL–LGR4–Gαq–β‐catenin pathway, which promotes the recruitment of osteoclast precursor cells and ultimately promotes the bone metastasis of breast cancer tumors.^12^ Upregulated expression of DKK1 in colorectal cancer can inhibit CD8^+^ T cells through the GSK3β–E2F1–T‐BET pathway and promote malignant progression of cancer cells.^27^ The DKK1–Wnt–β‐catenin signaling axis is negatively regulated by the NK4 gene in HNSCC, thus mediating the occurrence and development of HNSCC.^28^ DKK1 can also activate the Wnt‐planar cell polarity pathway to promote neuronal death in Alzheimer's disease.^29^ However, the mechanism of DKK1 and PNI remains unknown. We analyzed TCGA databases by KEGG analysis and found that PNI could upregulate the PI3K–AKT signaling pathway. Moreover, the PPI network and GEPIA2 tool showed a statistical correlation between DKK1 and Akt1. Akt1 is a serine/threonine‐protein kinase that regulates many processes, including proliferation and tumor growth.^30^ In pancreatic and esophageal cancers, DKK1 can bind to its receptor cytoskeleton‐associated protein 4 (CKAP4) and then activate the PI3K–AKT pathway to promote tumor growth.^11^ Akt can also be upregulated by CD74 and potentiate the secretion of GDNF to promote the PNI process in pancreatic ductal adenocarcinoma.^31^ There is still no report about the relationship and mechanism of Akt and PNI in HNSCC. In our study, we found that DKK1 can potentiate the expression of Akt1. Further study also found that an Akt inhibitor (MK2206) could reverse the migration abilities of SH‐SY5Y cells enhanced by DKK1‐overexpressing HNSCC cell lines. Therefore, we hypothesized that the PI3K–AKT signaling pathway may be downstream of DKK1 and then promote the PNI process.

However, there are still some limitations in our study. We initiated a preliminary exploration of mechanism of PNI, and the mechanisms of how DKK1 regulated PI3K–AKT signaling pathway were still unknown. In our future study, we will further verify the direct binding protein of DKK1 that could regulate PI3K–AKT signaling pathway and then promote the PNI process directly.

In conclusion, PNI was linked to poor clinical prognosis of HNSCC, and DKK1 may be an important biomarker for the PNI process. DKK1 was also upregulated in HNSCC and associated with PNI. DKK1 could promote the PI3K–AKT signaling pathway in tumor cells, and then could induce neuritogenesis and facilitate PNI. Our study implied that the DKK1–PI3K–AKT signaling pathway may be a potential predictive index and therapeutic target for HNSCC patients with PNI and that MK2206 may be a potential therapeutic target drug for HNSCC patients with high DKK1 expression.

## 4 MATERIALS AND METHODS

### 4.1 Patients and tissue samples

In this study, we collected HNSCC tissue samples. They were from 123 HNSCC patients at Sun Yat‐sen Memorial Hospital, Sun Yat‐sen University between 2014 and 2020. Detailed clinical and pathologic factors, including age, gender, TNM stage (UICC/AJCC 8th edition, 2018), survival state, treatment, and so forth, were obtained. The beginning of follow‐up duration was the complement of HNSCC treatment. The endpoints of follow‐up duration were OS time or DFS time. The median follow‐up time was 62.4 months [36–108 months] in our cohort. We also included 342 patients from TCGA database after excluding the patients with missing follow‐up data. There are still 77 patients from TCGA database without recurrence data. The median follow‐up time of 342 patients from TCGA database was 36.7 months [0–173 months].

The consent for the use of these clinical materials for research purposes was obtained. This study was approved by Institute Research Ethics Committee of Sun Yat‐sen Memorial Hospital, Sun Yat‐sen University, and followed the guidelines of the Helsinki Declaration.

### 4.2 Cell lines and cell cultures

Dulbecco's Modified Eagle′s Medium (DMEM) (Gibco) with 10% fetal bovine serum (Gibco) was used for culturing HEK293T and HNSCC cell lines including FaDu and TU686. Keratinocyte serum‐free medium (KSF, Invitrogen) was used for culturing normal squamous epithelial cells, which were isolated from noncancerous laryngopharyngeal tissues. All cells were cultured at 37°C with 5% CO_2_.

### 4.3 Data collection

In TCGA database, PNI^+^ and PNI^−^ had been evaluated and uploaded. We collected these data for the following analysis. In our cohort, PNI^+^ and PNI^−^ were scored by pathologists using H&E staining. The criteria of PNI^+^ nerve are invaded by or has at least 33% of its circumference surrounded by tumor cells.^32^

### 4.4 Immunohistochemistry

Detailed procedures of IHC were referred to the previous study,^33^ and the antibodies were anti‐DKK1 (1:400, ab109416, Abcam). For each slide, we chose three fields of vision for score. The immunoreactivity score (IRS) was performed according to the protocols of previous study.^33^ All IRS results were confirmed by at least two pathology experts through a double‐blind analysis. The median value 6 was the cutoff values of DKK1 IRS. Thus, the HNSCC patients were classified into two groups, which were high DKK1 expression groups (IRS ≥ 6.0) and low DKK1 expression groups (IRS < 6.0).

### 4.5 Immunofluorescence

Detailed protocols of IF were referred to our previous study.^34^ The antibodies and the concentration were anti‐DKK1 (1:400, ab109416, Abcam) and anti‐S100 (1:100, 66616‐1‐lg, Proteintech). Confocal images were acquired by a whole slide scanner (Vectra Polaris).

### 4.6 Establishment of stable cell lines

The full‐length human DKK1 opening reading frame (ORF) was cloned into pcDNA3.1^+^ (Clontech) and pLVX‐DsRed‐Monomer‐N1 (Clontech). Polyethyleneimine (PEI, YEASEN) were used to co‐transfect the lentiviral plasmids pLVX‐DsRed‐Monomer‐N1, psPAX2 (Clontech) and pMD2.G (Clontech). These lentiviral plasmids were co‐transfected at a mass ratio of 5:3.75:1.25 into HEK293T cells. After 48–72 h, the viral supernatants were collected and transduced with polybrene (YEASEN) to the cancer cells for 24 h. After the addition of 1 µg/mL of puromycin for a week, Western blot, real‐time PCR, and Elisa were performed to verify the success of the establishment of stable cell lines.

### 4.7 DRG culture

In this study, 6–8‐week‐old C57BL/6J mice were selected. Before surgery, mice were executed with a lethal dose of isoflurane. Mice were placed back up in the ultra‐clean cabinet. After sterilization by 75% ethanol, the dorsal skin was incised to expose the spine. The dorsal bone of the spine was removed to expose the spinal cord, and the dorsal root ganglion (DRG) could be dissected by gently removing the spinal cord and capsule. DRG were seeded in 12‐well plates in DMEM with 10% fetal bovine serum at 37°C in 5% CO_2_ for 30 min. HNSCC cells were added 1 mm from the DRG. Neurite extension was imaged after a week by microscope (Axio Observer Z1, Zeiss).

### 4.8 Western blot

Adherent cells were lysed by RIPA lysis buffer (CWBIO) containing protease inhibitors (CWBIO) on ice for 30 min. BCA kit (Thermo Fisher) was used for protein concentration determination. Detailed protocols of Western blot had been described previously.^34^ Primary antibodies in this study included anti‐DKK1 (1:1000, ab109416, Abcam), anti‐GAPDH (1:5000, ab8245, Abcam), anti‐pan‐Akt (1:1000, 4691S, Cell Signaling Technology), and anti‐Akt‐S473 (1:1000, 4060T, Cell Signaling Technology). The secondary antibodies included goat anti‐rabbit IgG H&L (HRP) (1:5000, ab6721, Abcam) and goat anti‐mouse IgG H&L (HRP) (1:5000, ab6789, Abcam). Images were captured by photo system (Mini Chemi 910, SinSage).

### 4.9 Real‐time PCR

TRIzol reagent (Invitrogen) was used to extract total RNA and cDNA. Detailed procedure of real‐time PCR was as described previously.^34^ The primers in the study are shown in Table S1.

### 4.10 ELISA

The cellular supernatants were put onto the surface of microplate wells (ThermoFisher Scientific) at 4°C overnight. The wells were blocked with 5% bovine serum albumin (BSA) at room temperature for 2 h. Then the anti‐DKK1 (100 ng/mL) was added to the wells for incubations at room temperature for 1 h. After washing for at least three times by PBS, goat anti‐rabbit IgG H&L (HRP) (1:12,000; ab6721, Abcam) was added to the wells for incubations at room temperature for 1 h. After the incubations of the substrates and stop solutions, the optical density (OD) was measured by spectrophotometer at a wavelength of 450 nm.

### 4.11 Transwell assay

The cells (3–5 × 10^4^ cells) were plated in DMEM without serum in the upper chambers of inserts (Corning) in a 24‐well plate. The bottom chambers were added with seeded HNSCC cells or 500 µL 10% DMEM as a chemo‐attractant. Detailed protocols of migration assay were performed, as described previously.^34^ All experiments were done in triplicate.

### 4.12 In vivo tumorigenesis and model of tumor sciatic neural invasion

Female BALB/c nude mice (4–5 weeks old) were injected subcutaneously with HNSCC cells resuspended in 100 µL of PBS containing 20% Matrigel. The length and width of the tumors were measured by Vernier calipers every 5 days. The mice were euthanased after 3 weeks. The tumors were collected, weighed, and paraffin embedded for IHC.

The procedure of the model of tumor sciatic neural invasion was performed according to the sciatic nerve invasion model of Deborde et al.^35^ Female nude mice (4–5 weeks old) were anesthetized. After fixation, the skin and subcutaneous tissues were cut, and the biceps femoris and gluteus maximus muscles were isolated, between which was the sciatic nerve. Tumor cells were injected. The grasping ability of the mice was dynamically observed. Tumors were collected after 2 weeks and paraffin embedded for IHC.

All the in vivo experiments were approved and supervised by the Animal Ethics Committee of Sun Yat‐sen University.

### 4.13 Statistical analysis

All the statistical analysis was performed by the Statistical Package for Social Sciences, version 25.0 (SPSS) or GraphPadPrism Version 8.0.2 (263). Either *χ* ^2^ test or Fisher's exact test was used to analyze the association of DKK1 or PNI with clinicopathological features. Two‐tailed Student's *t*‐test was used for differences among variables. To cultivate the significance of prognostic factors on OS or DFS, univariate and multivariate regression analysis was performed by the Cox proportional hazards model. Kaplan–Meier survival analysis was used for mapping survival curves. The survival differences between two groups were compared by the log‐rank test. When a *p*‐value was less than 0.05, the differences were considered statistically significant.

## AUTHOR CONTRIBUTIONS

Jingyi Wang conceived ideas, performed experiments, analyzed data, drafted manuscript, and administrated funding. Qianying Li performed experiments and analyzed data. Faya Liang collected the patients’ information and analyzed data. Xin Du analyzed data. Pan Song, Renhui Chen, and Taowei Wu collected the patients’ information. Qinglian Liu performed experiments. Xiaorong Lin administrated funding. Hai Hu, Ping Han, and Xiaoming Huang administrated funding and revised the manuscript. All authors have read and approved the final manuscript.

## CONFLICT OF INTEREST STATEMENT

The authors declare they have no conflicts of interest.

## ETHICS STATEMENT

Consent was taken for all HNSCC clinical samples to be used for research purposes. It was approved by Institute Research Ethics Committee of Sun Yat‐sen Memorial Hospital, Sun Yat‐sen University, and followed the guidelines of the Helsinki Declaration (approval ID: SYSKY‐2023‐111‐01). All the in vivo experiments were approved and supervised by the Animal Ethics Committee of Sun Yat‐sen University (approval ID: SYSU‐IACUC‐2023‐000503).

## Supporting information

Supporting Information

## Data Availability

The datasets used and/or analyzed during the current study are available from public database or the corresponding author upon reasonable request. The sequence data and clinical information are publicly available from the TCGA database (https://www.cbioportal.org/).
