# Supplementary material for: Dickkopf‐1 drives perineural invasion via PI3K–AKT signaling pathway in head and neck squamous cancer
Source: MedComm (2020). 2024 Mar 22;5(4):e518. doi: 10.1002/mco2.518 (PMC10959454; doi:10.1002/mco2.518)
Supplement: Supplementary file 1 — Supporting Information [file MCO2-5-e518-s001.docx]

**Title：****Dickkopf-1 Drives Perineural Invasion via PI3K-AKT signaling pathway in** **Head and Neck Squamous Cancer**

**Running title：DKK1 Drives Perineural Invasion in HNSCC**

Jingyi Wang^1,2†^, Qianying Li^1,2†^, Faya Liang^1,2^, Xin Du^3^, Pan Song ^1,2^, Taowei Wu^1,2^, Renhui Chen^1,2^, Xiaorong Lin^4^, Qinglian Liu^3^, Hai Hu^3^, Ping Han^1,2*^, Xiaoming Huang^1,2*^

† Both authors have contributed equally to this work.

1 Department of Otolaryngology-Head and Neck Surgery, Sun Yat-sen Memorial Hospital, Sun Yat-sen University, Guangzhou, China.

2 Guangdong Provincial Key Laboratory of Malignant Tumor Epigenetics and Gene Regulation, Guangzhou, China

3 Department of Oncology, Sun Yat-Sen Memorial Hospital, Sun Yat-sen University, Guangzhou, China.

4 Diagnosis and Treatment Center of Breast Diseases, Shantou Central Hospital, Shantou, China.

*Correspondence to: Xiaoming Huang and Ping Han.

* Correspondence:

Corresponding author:

*Xiaoming Huang, Department of Otolaryngology-Head and Neck Surgery, Sun Yat-sen Memorial Hospital, Sun Yat-sen University, 107 Yanjiang West Road, Guangzhou, China 510120

E-mail: hxming@mail.sysu.edu.cn

*Ping Han, Department of Otolaryngology-Head and Neck Surgery, Sun Yat-sen Memorial Hospital, Sun Yat-sen University, 107 Yanjiang West Road, Guangzhou, China 510120

E-mail: hanping5@mail.sysu.edu.cn

# Supplementary Figure

**
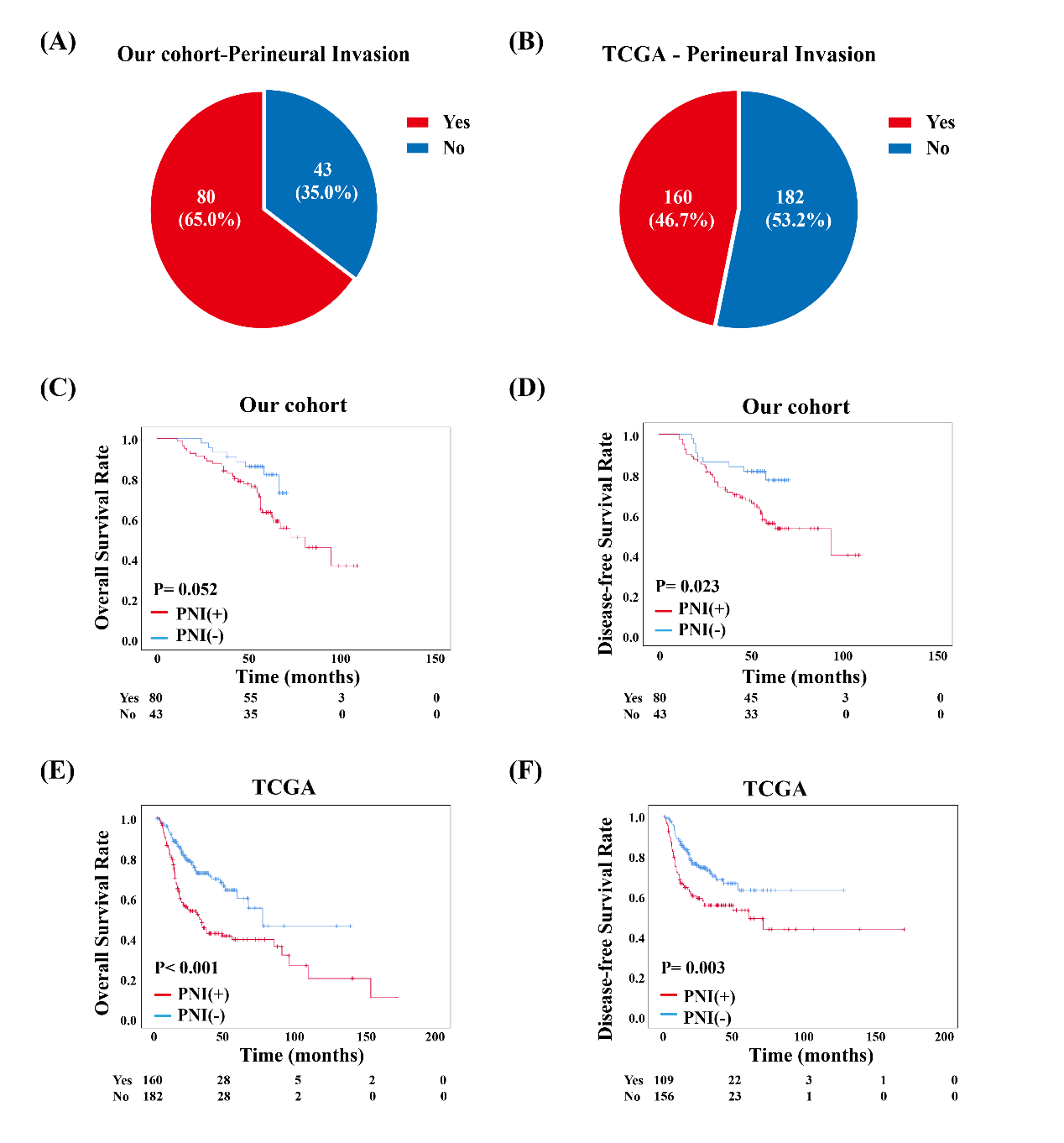
**

**Figure S1. PNI correlates with poor prognosis of HNSCC**

A-B. The Proportion of PNI among HNSCC patients in our cohort (A) and TCGA databases (B).

C-D. Kaplan–Meier survival analysis showing the OS(C) and DFS(D) of HNSCC patients with or without PNI among HNSCC patients in our cohort (log-rank test).

E-F. Kaplan–Meier survival analysis showing the OS(E) and DFS(F) of HNSCC patients with or without PNI among HNSCC patients in TCGA database (log-rank test).

**
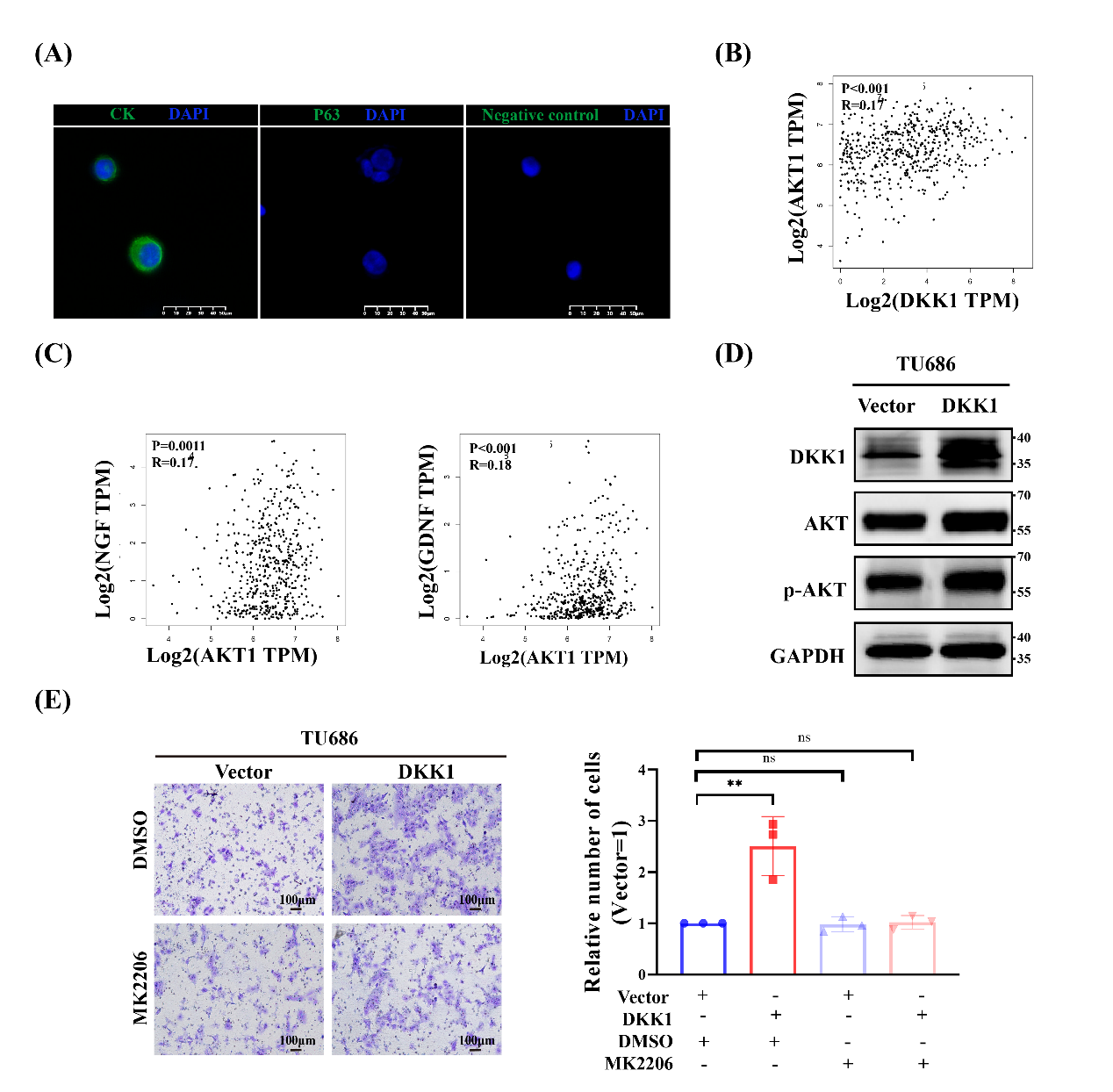
**

**Figure S2. DKK1 regulates HNSCC cell migration and PNI through PI3K-Akt signaling pathways**

A. Immunofluorescence staining Cytokeratin, P63 and negative control in normal squamous epithelial cells. Green- Cytokeratin, P63, negative control; Blue- DAPI.

B-C. The correlation between Akt1 and DKK1/NGF/GDNF was analyzed by GEPIA tools.

D. Western blot showing DKK1 promoting the expression of AKT and p-AKT(TU686).

E. Representative images of migration assays of vector or DKK1-overexpressing HNSCC cell lines (TU686) after incubation with DMSO or MK2206 (10 nM). The results are from three different experiments. The number of migrated cells per field of view was plotted.

# Supplementary Table

**Table S1. Primer sequences for real-time PCR**

| **Target** | **Sense (5’-3’)** | **Antisense (5’-3’)** |
| --- | --- | --- |
| **DKK1** | GTGCAAATCTGTCTCGCCTG | GCACAGTCTGATGACCGGAG |
| **Akt1** | GCCCGAAGACGGGAGC | CTCACGTTGGTCCACATCCT |
| **β-actin** | AGTCATTCCAAATATGAGATGCGTT | TGTGGACTTGGGAGAGGACT |
